# Primary Care Perspectives of Decision‐Making Assets for African American Dementia Dyads

**DOI:** 10.1002/alz70858_104057

**Published:** 2025-12-24

**Authors:** Kalisha Bonds Johnson, Daum Chung, Gaea Daniel, Olachi Unaka, Kenneth Hepburn, Molly Campbell, Theodore M Johnson

**Affiliations:** ^1^ Emory University, Atlanta, GA, USA; ^2^ Linfield University, Portland, OR, USA

## Abstract

**Background:**

Eighty two percent of primary care providers (PCPs) report they are the frontline providers of Alzheimer's disease and related dementias (ADRD) care in the United States. African American dementia dyads (i.e., person living with ADRD and family care partner) want PCPs with experience providing quality care to African American persons living with ADRD. Despite concerns of pervasive distrust in the healthcare system by African American dementia dyads, there may be facilitators that support their health care decision making.

**Method:**

We conducted a qualitative study exploring facilitators to health care decision making for African American dementia dyads. A purposive sample of 20 primary care providers (i.e., physicians, nurse practitioners) and care team members (i.e., nurses, social workers) completed semi‐structured teleconference interviews guided by the Black Family Social‐Ecological Model. Qualitative directed content analysis was used to analyze the data.

**Result:**

Three themes were identified (1) systemic healthcare assets, (2) relational health care assets, and (3) providers’ and care team members’ individual assets. The themes diverged to yield ten categories and four subcategories. The ten categories were (1) dementia‐specific care, (2) culturally specific care, (3) essential team members, (4) links to community resources, (5) providers’ or care team members’ approaches to communication, (6) recognition of health disparities, (7) cordial understanding, (8) providers’ and care team members’ self‐work, (9) extended follow‐up and proactive support, and (10) substantiate feelings and efforts. Initial consultation with the person living with ADRD and initial consultation with the dyad/family are the two subcategories of providers’ or care team members’ approaches to communication. Providers’ or care team members’ background and providers’ or care team members’ roles on the team are subcategories of providers’ and care team members’ self‐work.

**Conclusion:**

Providers and care team members expressed the importance of potential assets at multiple ecological levels. Healthcare systems, providers, and care team members are responsible in providing quality care to African American dementia dyads. Thus, interventions may need to focus on multiple levels to meet the health care needs of African American persons living with ADRD.

